# Supplementary material for: Glial-Cell-Line-Derived Neurotrophic Factor Promotes Glioblastoma Cell Migration and Invasion via the SMAD2/3-SERPINE1-Signaling Axis
Source: Int J Mol Sci. 2024 Sep 23;25(18):10229. doi: 10.3390/ijms251810229 (PMC11432670; doi:10.3390/ijms251810229)
Supplement: Supplementary file 1 [file ijms-25-10229-s001.zip › Supplementary materials.pdf]

Supplementary Figures

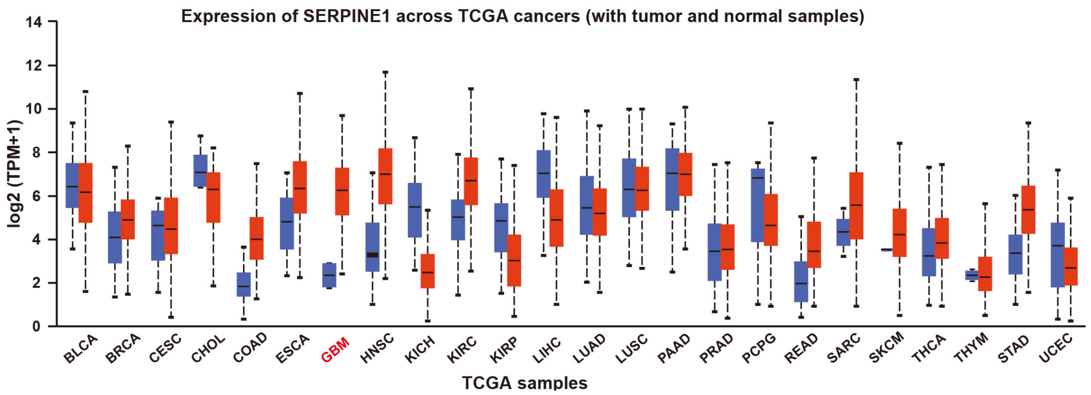

Figure S1. The mRNA levels of SERPINE1 were analyzed between multiple human tumors and corresponding normal tissues using UALCAN database.

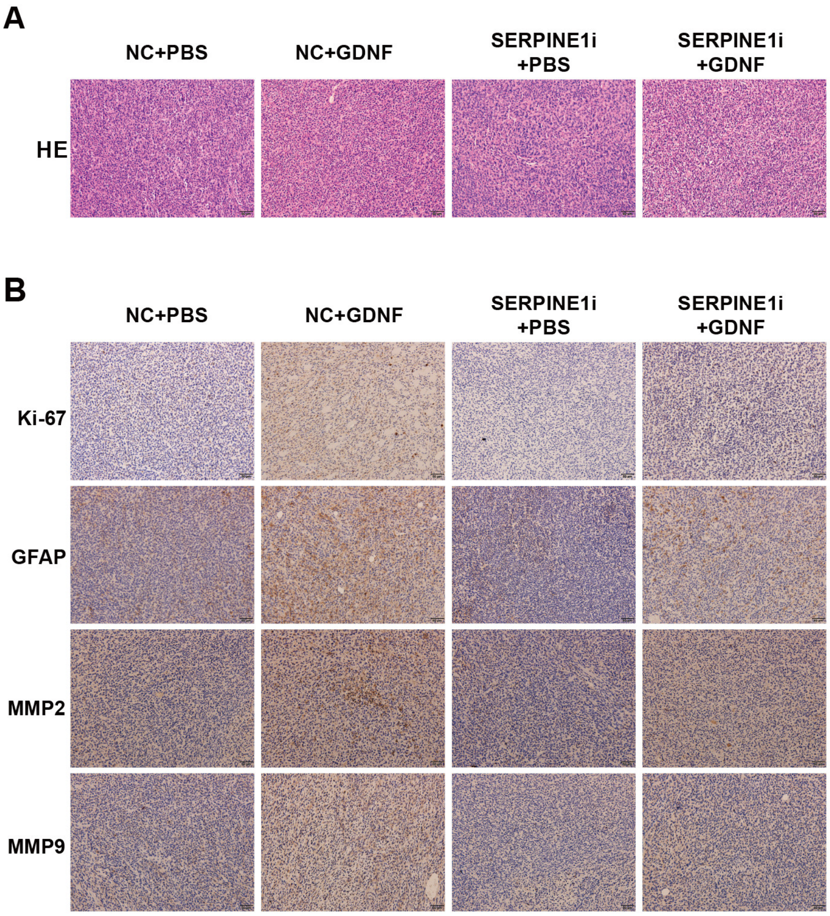

Figure S2. GDNF accelerates GBM growth *in vivo*. (A) HE staining of tumor tissues. (B) Immunohistochemistry was performed to detect the levels of ki-67, GFAP, MMP2 and MMP9. Bar = 50 μm. n = 5.

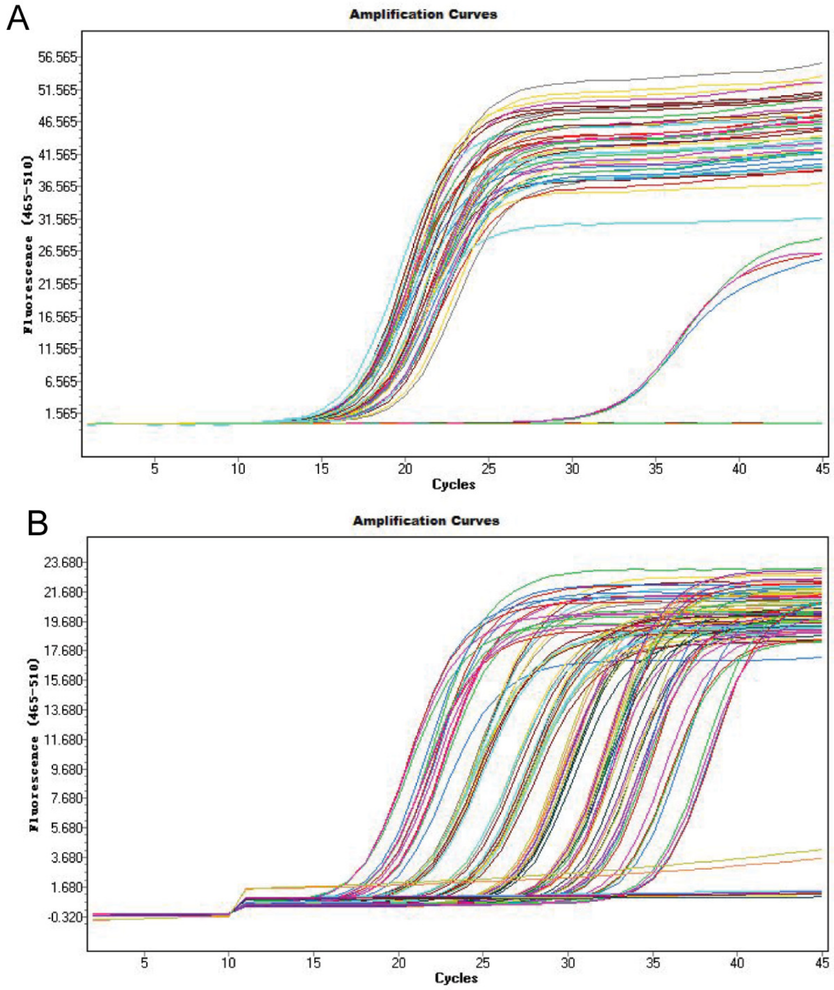

Figure S3. The amplificaion curves of SERPINE1 primers in U251 (A) and C6 (B) cells.

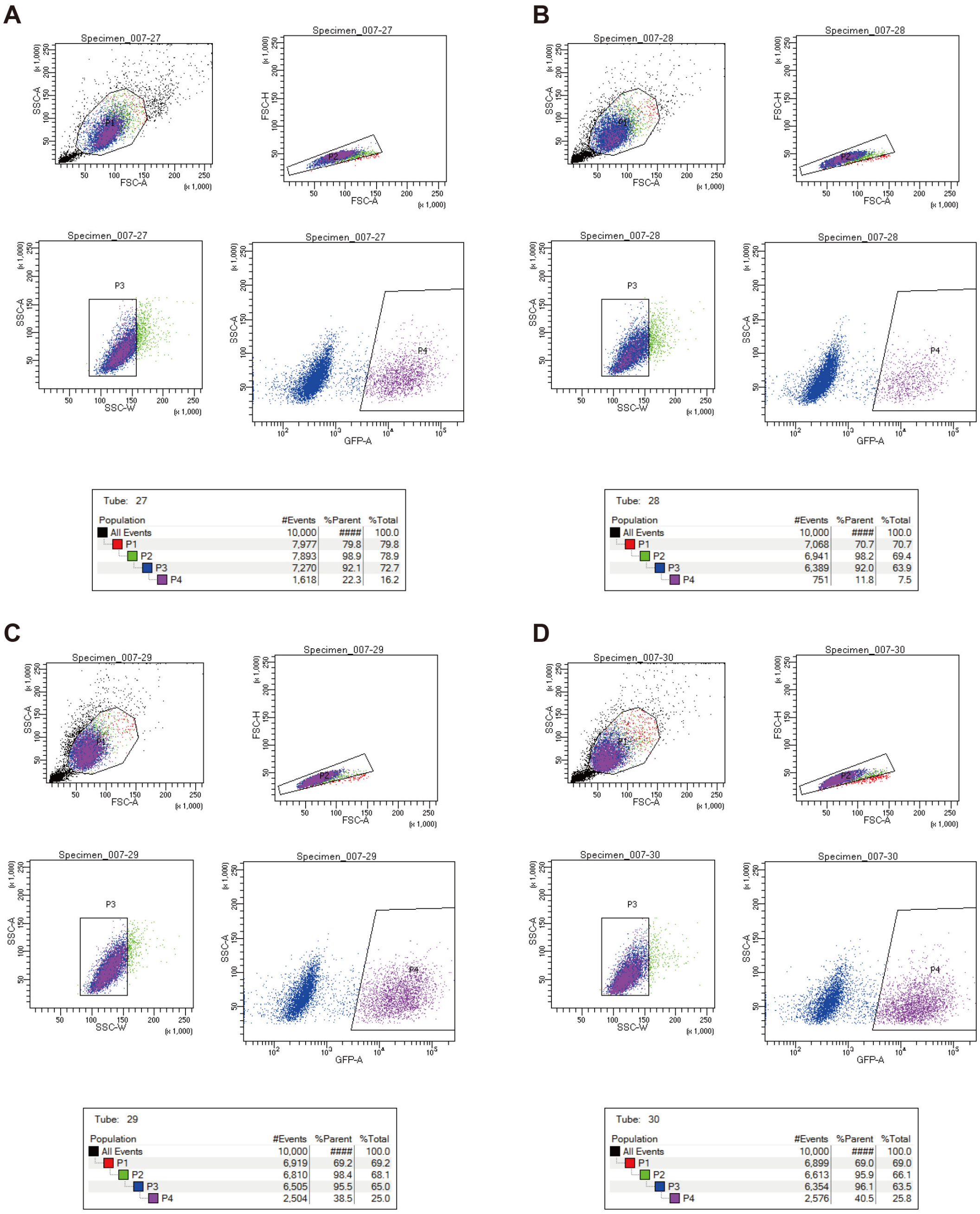

Tube: 27

Population	#Events	%Parent	%Total
All Events	10,000	####	100.0
P1	7,977	79.8	79.8
P2	7,893	98.9	78.9
P3	7,270	92.1	72.7
P4	1,618	22.3	16.2

Tube: 28

Population	#Events	%Parent	%Total
All Events	10,000	####	100.0
P1	7,068	70.7	70.7
P2	6,941	98.2	69.4
P3	6,389	92.0	63.9
P4	751	11.8	7.5

Tube: 29

Population	#Events	%Parent	%Total
All Events	10,000	####	100.0
P1	6,919	69.2	69.2
P2	6,810	98.4	68.1
P3	6,505	95.5	65.0
P4	2,504	38.5	25.0

Tube: 30

Population	#Events	%Parent	%Total
All Events	10,000	####	100.0
P1	6,899	69.0	69.0
P2	6,613	95.9	66.1
P3	6,354	96.1	63.5
P4	2,576	40.5	25.8

Figure S4. C6 cells were sorted by FACS based on SERPINE1 knockdown. (A) Negative control group. (B) RNAi-1 group. (C) RNAi-2 group. (D) RNAi-3 group.

Supplementary Tables

Table S1: Primer sequences for qRT-PCR.

Gene	Forward sequence	Reverse sequence
Human	5'-GCGTCTTCCTCCACAGCCATTC-3'	5'-TGTCTCTGTTGGATTGTGCCGAAC-3'
Rat SERPINE1	5'-GCGTCTTCCTCCACAGCCATTC-3'	5'TGTCTCTGTTGGATTGTGCCGAAC-3'
Human GAPDH	5'-AGAAGGCTGGGGCTCATTTG-3'	5'-AGGGGCCATCCACAGTCTTC-3'
Rat GAPDH	5'-CAGTGCCAGCCTCGTCTCAT-3'	5'-AGGGGCCATCCACAGTCTTC-3'

Table S2: siRNA sequences.

siRNA name	Species	Sequences
si-SERPINE1-#1	Human	Sense: CCCUCCGGCAUCUGUACAATT<br>Antisense: UUGUACAGAUGCCGGAGGGTT
si-SERPINE1-#2	Human	Sense: CCGGAGCACGGUCAAGCAATT<br>Antisense: UUGCUUGACCGUGCUCCGGTT
si-SERPINE1-#3	Human	Sense: GCCACUGGAAAGGCAACAUTT<br>Antisense: AUGUUGCCUUUCCAGUGGCTT
si-SMAD2	Rat	Sense: GCCGAGUGCCUAAGUGAUATT<br>Antisense: UAUCACUUAGGCACUCGGCTT
si-SMAD3	Rat	Sense: GCUUUGAGGCUGUCUACCATT<br>Antisense: UGGUAGACAGCCUCAAAGCTT
