# Supplementary figures and images for: Glial-Cell-Line-Derived Neurotrophic Factor Promotes Glioblastoma Cell Migration and Invasion via the SMAD2/3-SERPINE1-Signaling Axis
Source: Int J Mol Sci. 2024 Sep 23;25(18):10229. doi: 10.3390/ijms251810229 (PMC11432670; doi:10.3390/ijms251810229)

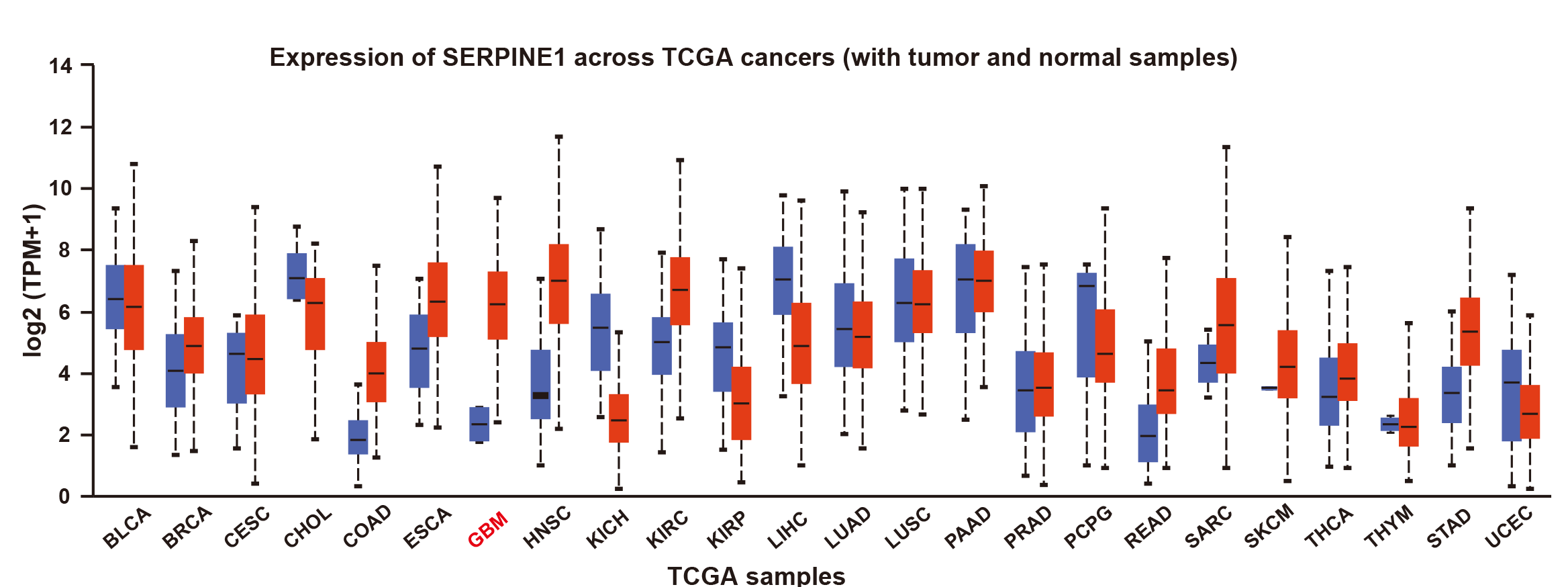

Supplement: Supplementary file 1 [file ijms-25-10229-s001.zip › Figure S1.tif]

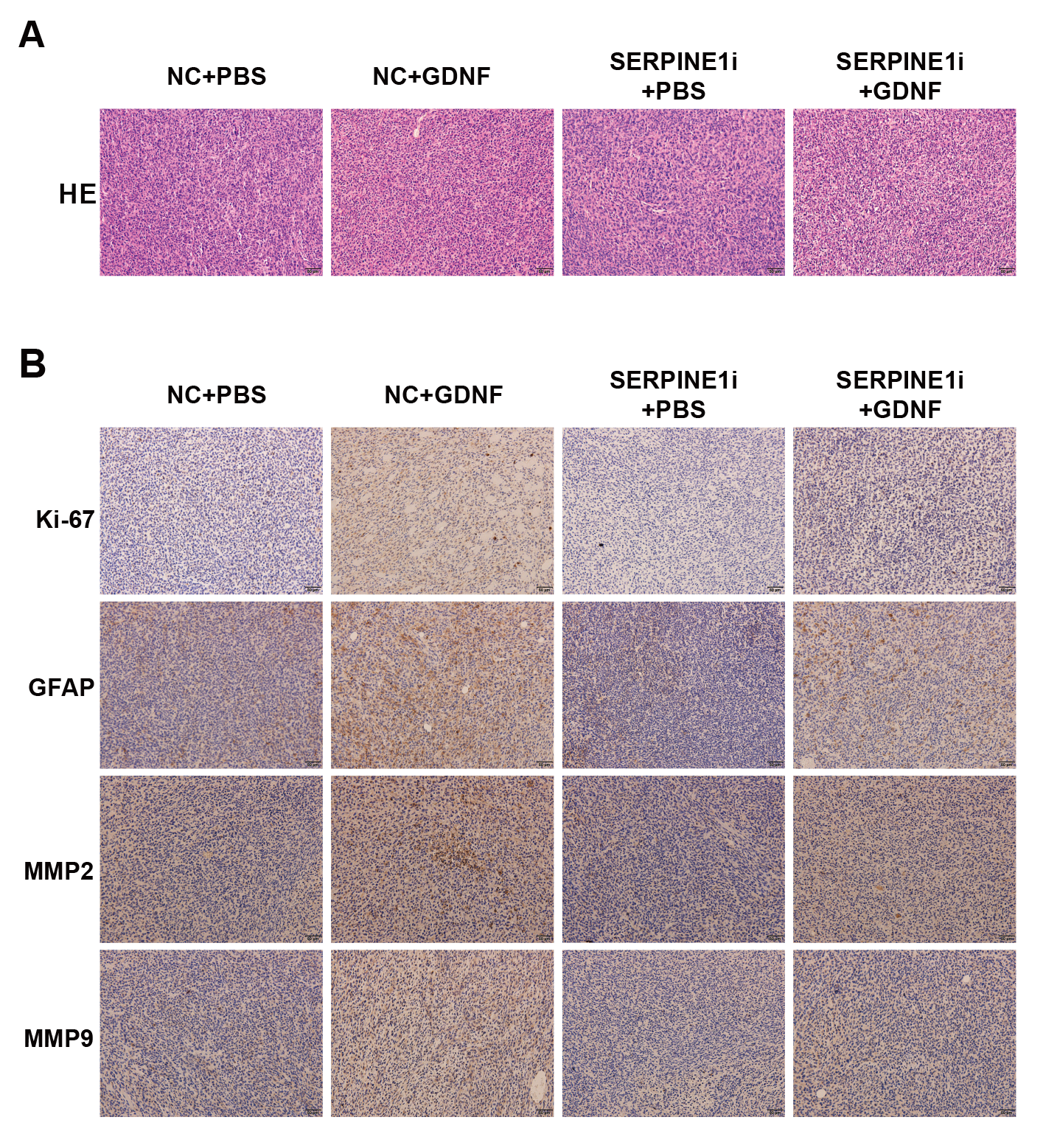

Supplement: Supplementary file 1 [file ijms-25-10229-s001.zip › Figure S2.tif]

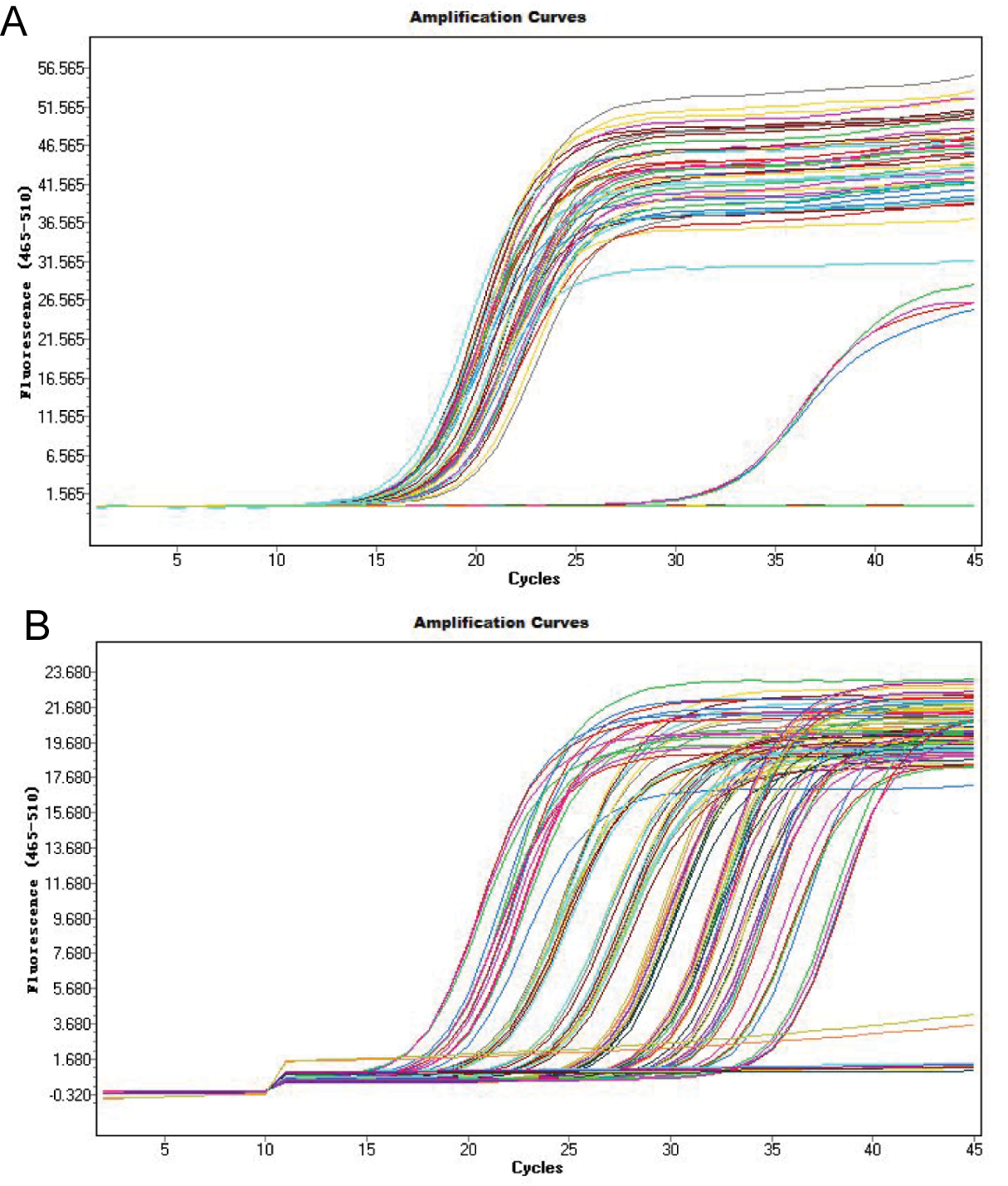

Supplement: Supplementary file 1 [file ijms-25-10229-s001.zip › Figure S3.tif]

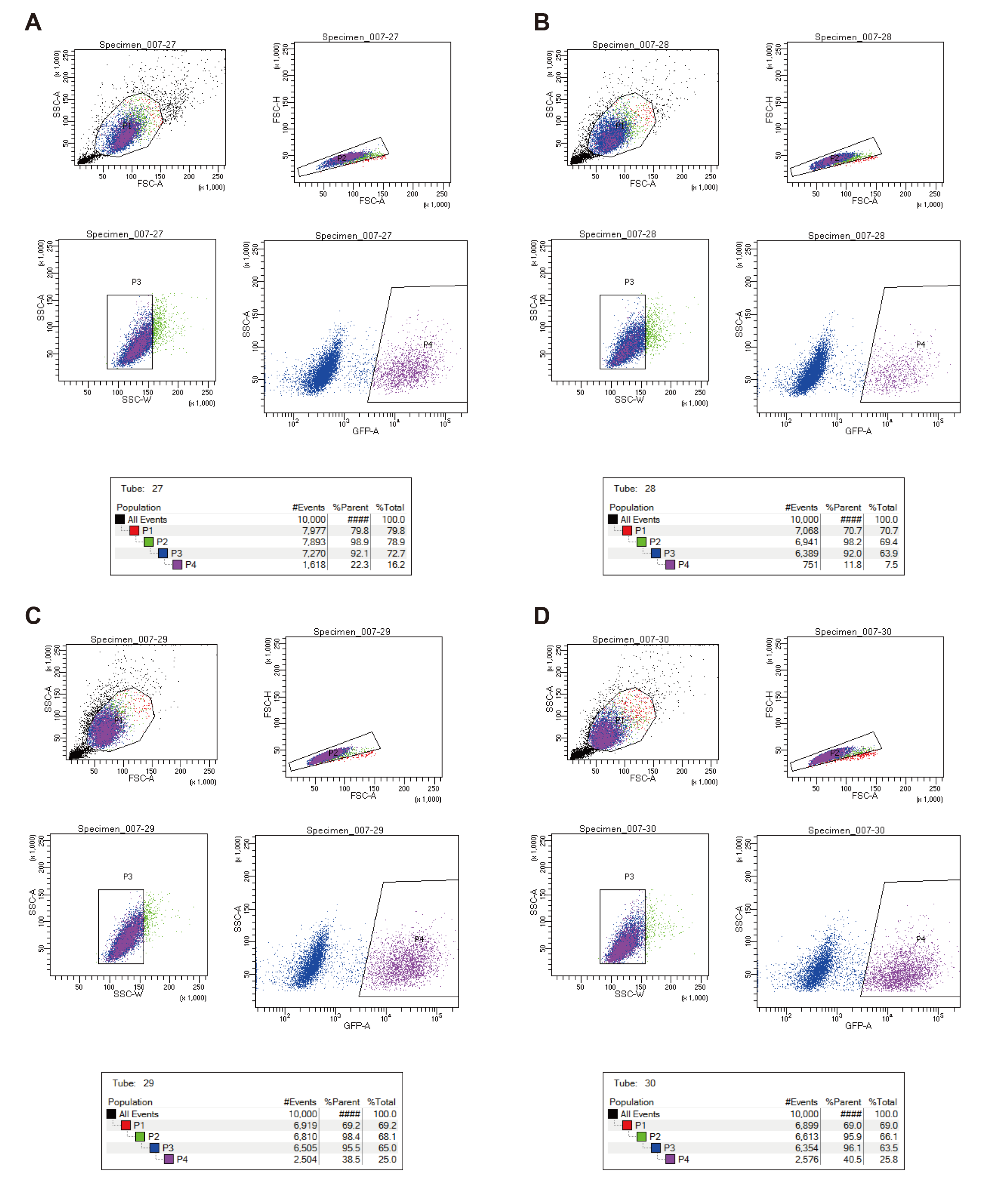

Supplement: Supplementary file 1 [file ijms-25-10229-s001.zip › Figure S4.tif]
